# Supplementary figures and images for: DNA metabarcoding reveals the dietary profiles of a benthic marine crustacean, Nephrops norvegicus
Source: PLoS One. 2023 Nov 1;18(11):e0289221. doi: 10.1371/journal.pone.0289221 (PMC10619785; doi:10.1371/journal.pone.0289221)

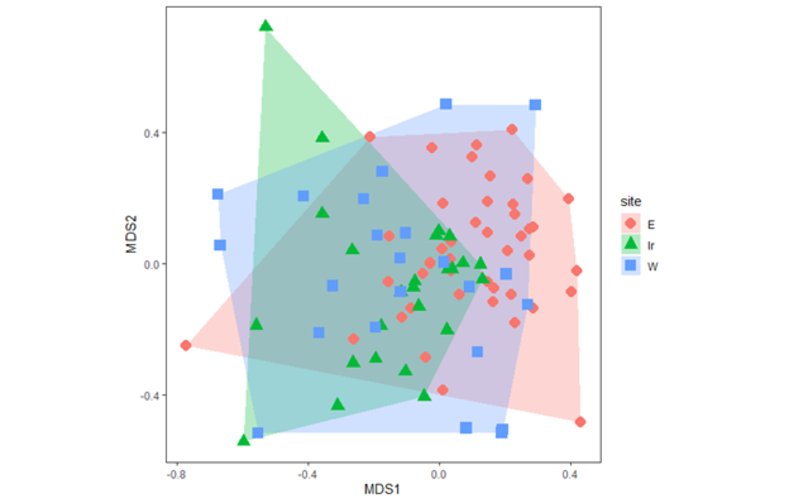

Supplement: S1 Fig — (TIF) [file pone.0289221.s001.tif]
