# Supplementary material for: DNA metabarcoding reveals the dietary profiles of a benthic marine crustacean, Nephrops norvegicus
Source: PLoS One. 2023 Nov 1;18(11):e0289221. doi: 10.1371/journal.pone.0289221 (PMC10619785; doi:10.1371/journal.pone.0289221)
Supplement: Attachment — Submitted filename: PLOS Review Norwegian Lobsters.docx [file pone.0289221.s002.docx]

Review – POME-D-23-04632 – DNA metabarcoding reveals the dietary profiles of a benthic marine crustacean Nephrops norvegicus

**Overview:**

Overall, this is a nice informative manuscript that is using new techniques to provide information on the diet of an important crustacean. While the manuscript provides good data, I think there are some edits that would greatly improve it, particularly in the discussion and introduction where more details could be added. Major and minor comments are listed below.

**Major comments:**

Check overall reference style for PLOS submitting. I believe they use numbers to identify in text citations. Also be sure to be consistent, in some places in the in text citations you italicize et al. and in other places you don’t. Please confirm the style on the PLOS style guide.

Line 127 – What defines the East and the West of the North Sea? Do you have specific coordinates that separate them? A map here would be helpful to include as a figure to show the breakdown of where the samples are coming from geographically.

Line 177-179 I am a little unclear here, is this the species diversity of the sites that were sampled and what you would expect to find there or the species diversity of the organisms found as prey inside of the lobsters? If it is species diversity at the overall site, where did that data come from?

Line 217-219 Be careful of interpretation in the results section. Here, I would just state that the rarefaction curves failed to reach saturation for each group. Save the part of the sentence that says this implies increased sampling… for the discussion section of the paper.

Line 234-235 How were these percentages calculated? The supplemental table and figure help, but don’t fully explain where those results come from. I would also mention the low sampling resolution in the discussion, not hear in the results section.

White beaked dolphins were found in the prey items of the lobster? Would you expect this that they would be prey upon dolphins, or is it material they are scavenging? Is there other evidence for mammal material in lobsters or other crustaceans? Please elaborate in the discussion about finding mammal DNA in the gut contents.

Line 286-287 What constitutes a high rate of infection? Are their known concentrations of the diatom in the area? Is it high compared to the relative abundance of the diatom or is it high compared to other available prey items that were found from barcoding?

Line 321-322 If there are reports of cannibalism, why assume it is host contamination? Because of the higher value (98%)? Please elaborate more on why it was appropriate to treat it as host contamination since there is evidence of cannibalism in the species.

**Minor comments:**

Line 59: Should faeces be feces?

Line 58-62 Consider switching the order of these two sentences.

Line 106 Add commas around “especially by females”

Line 106- When is the long breeding season? Does it correspond to when the samples were collected – see if they have a discussion on sex related and timing related prey identification

Line 107 Did the Santana et al. 2020 paper find this on samples collected during the long breeding time? It might be nice to include what time of year their samples came from for even further context about when they suspension feed.

Line 118-120 This sentence seems to have two separate ideas and is a little bit hard to follow. Can you please revise, perhaps break it into two separate sentences.

Line 133 Can you provide manufacturer details beyond just the website? Location of company? Same for BIOO Scientific on line 152.

Line 182 Before using MOTU, please define what that acronym means.

Line 190 Is that the same version of R as previously mentioned on line 183?

Line 204 What are the positive and negative controls?

Line 211 Can remove “With a modest number of remaining reads” and start the sentence with “We considered data…”.

Line 220 Add ‘and’ between 49% and 46%
