# Supplementary material for: DNA metabarcoding reveals the dietary profiles of a benthic marine crustacean, Nephrops norvegicus
Source: PLoS One. 2023 Nov 1;18(11):e0289221. doi: 10.1371/journal.pone.0289221 (PMC10619785; doi:10.1371/journal.pone.0289221)
Supplement: Attachment — Submitted filename: Reviewer_comments_R2.docx [file pone.0289221.s004.docx]

Thank you for re-submitting your manuscript to PLOS ONE. Both of the prior reviewers have made a second evaluation, and one of these reviewers has made a few additional suggestions that are primarily editorial and do not affect the scientific value of the contribution, but I agree will make the contribution more scientifically sound. Therefore, I ask you to consider their suggestions as a final step to improve the manuscript and make it acceptable for publication. Taking into account these suggestions, please submit a revised version of the manuscript that addresses these final points raised during the review process.

**We have now evaluated the reviewers' comments (as per below) and believe the points raised are now satisfactory. In light of the review process, we acknowledge the reviewers anonymously for their comments.**

Comments to the Author

Reviewer #1: Thank you for the chance to review this resubmission. I have a few minor editorial comments that should be addressed:

- The in-text citations need to be adjusted. There are unnecessary parentheses, and the number order is inconsistent. For example, [(46), (44)] should be [44, 46]. Please correct this throughout the manuscript.

**We have made corrections with citations enclosed in square brackets [].**

- I recommend abbreviating the site names for the North Sea. East North Sea to ENS and West North Sea to WNS. The way it is written at times makes it seem like you could be referring to a "West Sea". For example, "the West and Irish Sea". There also several instances throughout where a direction (West of Ireland) is capitalized and does not need to be.

**We have taken this suggestion into account and corrected the names to North Sea East (NS-East) and North Sea West (NS-West).**

- Species should be italicized in the references.

**Corrected.**

- When referencing a specific paper in the text such as Santana et al. 2020, leave out the year and just insert the citation number. "Santana et al. [23] showed that...." See line 102, but there are at least one or two other occurrences.

**Corrected.**

- Line 100: put a period after "i.e., suspension feeding". Then start a new sentence "Suspension feeding is thought to be used especially by females..."

**Corrected.**

- Line 424: Should be "Southern Ocean" not "Antarctica ocean"

**Corrected.**

- Figure S2 was removed entirely rather than brought into the manuscript as was noted in a response to a previous comment. Was this intentional? I can't access the previous version or see tracked changes but believe the figures are the same as the original submission.

**This figure was implemented into Figure 2. It is cited as Figure 2B.**

- In order to address concerns raised by both reviewers, it may also be useful to add a statement in the conclusion that suggests further research is needed to explore the role of some of the "secondary" or unexpected prey items found in the Nephrops diet in greater detail.

**We have added a statement into the final sentence.**

Reviewer #2: All of my original concerns have been addressed, and this paper should be considered for acceptance.

**We thank the reviewer for their thorough evaluation and positive feedback.**
